# Supplementary material for: ReCo: automated NGS read-counting of single and combinatorial CRISPR gRNAs
Source: Bioinformatics. 2023 Jul 22;39(8):btad448. doi: 10.1093/bioinformatics/btad448 (PMC10400375; doi:10.1093/bioinformatics/btad448)
Supplement: btad448_Supplementary_Data [file btad448_supplementary_data.zip › Supplementary Figures.docx]

**Supplementary Figures**

**Supplementary Figure S1:** ReCo’s functional components are organized in a set of classes. The arrows and circled numbers 1 to 3 represent the initialization process. The black arrows and the circled number 4 represent the logical flow of processing a Single-end read sample (SingleSample). A Paired-end read sample (PairedSample) is processed analogously, for clarity the arrows are omitted. The Config class provides the configuration for ReCo and can be adjusted, the external tools cutadapt and bowtie are required to be available from the system path. The graph was created with BioRender.com.

**Supplementary Figure S2:** The ReCo plot panel visualizes the gRNA distribution of single-end and paired-end read samples. Here, a plasmid DNA preparation of the Brunello library [(Doench *et al.*, 2016)](https://paperpile.com/c/p05ue2/CCRf) was sequenced according to Spahn et al., 2017. The title of the plot contains the sample name, the number of expected reads, and the number of observed reads. **(a)** A stacked bar chart indicates the ratio of aligned (green color), not aligned (red color), and not trimmed (light blue color) reads. **(b)** A box plot displays the distribution of read counts on a logarithmic scale, the expected and observed mean read counts are highlighted with horizontal dashed lines in gray and red color, respectively. **(c)** The log-transformed distribution of read counts per gRNA, sorted increasingly. **(d)** A cumulative plot of gRNA abundance. The example library has an area under the curve (AUC) of 0.73. Out of a total of 77,441 gRNA sequences, 76,372 sequences were found, indicating that the library contains 98.62% of all intended gRNAs. For comparison, the cumulative distribution of an ideal, uniformly distributed library is plotted as a black diagonal line and has an AUC of 0.5. **(e)** A histogram of gRNA abundance and overlay of the corresponding density plot shows the skew of the library distribution. The 10 and 90 percentiles are indicated with vertical dashed lines, and the distribution skew of the library is 13.71.
