# Supplementary figures and images for: ReCo: automated NGS read-counting of single and combinatorial CRISPR gRNAs
Source: Bioinformatics. 2023 Jul 22;39(8):btad448. doi: 10.1093/bioinformatics/btad448 (PMC10400375; doi:10.1093/bioinformatics/btad448)

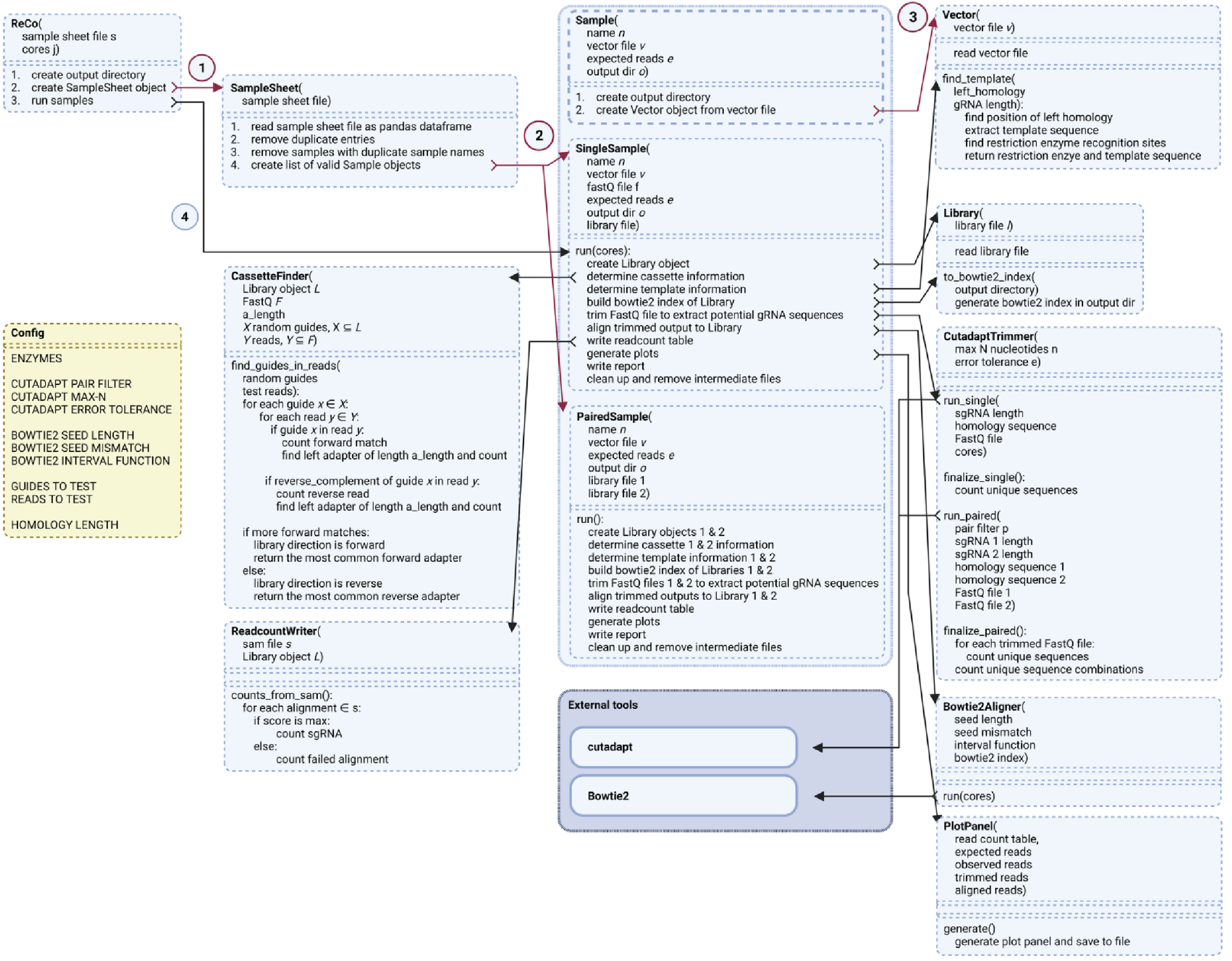

Supplement: btad448_Supplementary_Data [file btad448_supplementary_data.zip › Supplementary_Figure_S1.png]

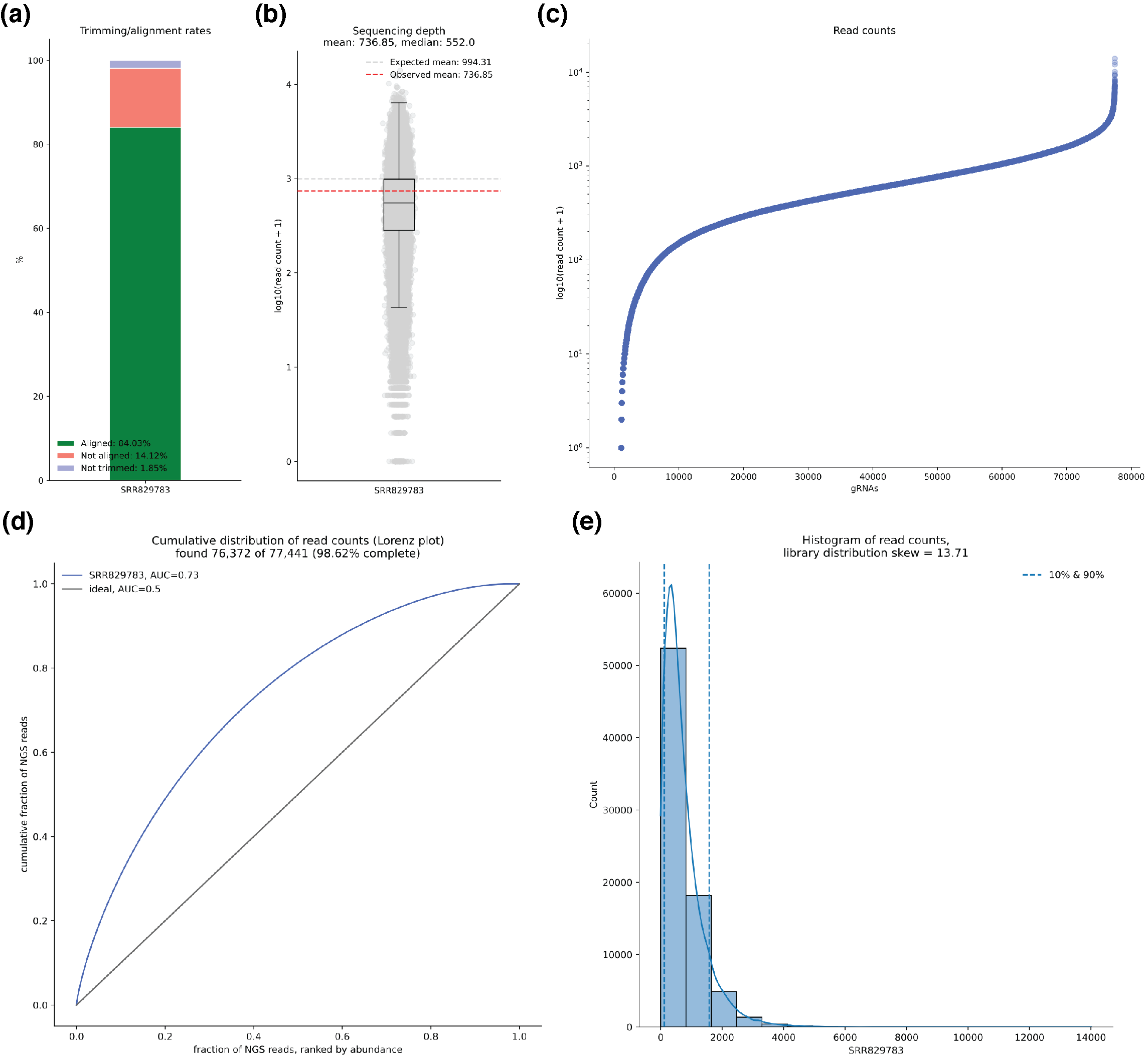

Supplement: btad448_Supplementary_Data [file btad448_supplementary_data.zip › Supplementary_Figure_S2.png]
